# Cognitive control network connectivity differentially disrupted in treatment resistant schizophrenia

**DOI:** 10.1016/j.nicl.2021.102631

**Published:** 2021-03-17

**Authors:** Charlotte M. Horne, Lucy D. Vanes, Tess Verneuil, Elias Mouchlianitis, Timea Szentgyorgyi, Bruno Averbeck, Robert Leech, Rosalyn J. Moran, Sukhwinder S. Shergill

**Affiliations:** a Institute of Psychiatry, Psychology and Neuroscience, King’s College London, De Crespigny Park, London SE5 8AF, United Kingdom; b Laboratory of Neuropsychology, National Institute for Mental Health Bethesda, BETHESDA, MD 20814, USA

**Keywords:** Schizophrenia, Functional MRI, Glutamate, Treatment resistance, Dynamic causal modelling

## Abstract

Antipsychotic treatment resistance affects a third of people with schizophrenia and the underlying mechanism remains unclear. We used an fMRI emotion-yoked reward learning task, allied to prefrontal cortical glutamate levels, to explain the role of cognitive control in differentiating treatment-resistant from responsive patients. We investigated how reward learning is disrupted at the network level in 21 medicated treatment-responsive and 20 medicated treatment-resistant patients with schizophrenia compared with 24 healthy controls (HC). Dynamic Causal Modelling assessed how effective connectivity between regions in a cortico-striatal-limbic network is disrupted in each patient group compared to HC. Connectivity was also examined with respect to symptoms, salience and anterior cingulate (ACC) glutamate levels measured from the same region of the ACC. We found that ACC connectivity differentiated these patient groups, with responsive patients exhibiting increased top-down connectivity from ACC to sensory regions and reduced ACC drive to the striatum, while resistant patients showed altered connectivity within the ACC itself. In these resistant patients, the ACC drive to striatum was positively correlated with their symptom severity. ACC glutamate levels were found to correlate with ACC control over sensory regions in responsive patients but not in resistant patients. We suggest a central non-dopaminergic impairment that impacts cognitive control networks in treatment-resistant schizophrenia. This impairment was associated with disrupted reward learning and could be underpinned by aberrant glutamate function. These findings should form the focus of future treatment strategies (e.g. glutamatergic targets and giving clozapine earlier) in resistant patients.

## Highlights

• Mechanisms underlying treatment-resistant schizophrenia are unclear.
• Effective connectivity within cortico-striatal network differentially disrupted in resistant patients.
• Resistance associated with lack of top-down control and aberrant glutamate function.
• We suggest a subtype of schizophrenia with distinct neurobiological mechanism.
• Results are important for guiding treatment strategies and developing new drugs.

## 1 Introduction

Approximately one third of patients with schizophrenia fail to respond to antipsychotic medication, termed ‘treatment resistance’ (Lindenmayer, 2000). Almost all currently licensed antipsychotics block dopaminergic D2 receptors in the brain (Coppens et al., 1991). However, resistance occurs despite adequate dopaminergic blockade (Wolkin et al., 1989) and is associated with significantly poorer outcomes (Marshall et al., 2005). Clozapine is currently the only licensed antipsychotic that has some added benefit for treating treatment-resistant patients although 60% will still not respond (Siskind et al., 2017). Clozapine’s mechanism of action in the brain is unclear but likely targets other mechanisms beyond D2 receptor antagonism in the striatum (Potkin et al., 2020). Therefore, identifying the underlying mechanisms for resistance is essential for guiding future therapeutic strategies.

Aberrant dopamine signalling in schizophrenia has been associated with a ‘drowning out’ of reward prediction errors (RPE) (difference between expected and actual reward outcomes) (Howes and Kapur, 2009) and the consequent misattribution of salience to irrelevant stimuli in the environment (Heinz and Schlagenhauf, 2010, McCutcheon et al., 2020). This contributes to impaired reinforcement learning; the ability to learn which stimuli are correctly associated with reward using sequential decision-making (Murray et al., 2008, Kapur, 2003). In turn, patients rely to a greater extent on strong prior experiences for decision-making (top-down control) in order to compensate for more unreliable sensory perception (Friston et al., 2016). This impacts on the ability to efficiently update their beliefs which in turn could result in the classical positive symptoms of schizophrenia (Powers et al., 2017, Corlett et al., 2011) – hallucinations, or perceptual experiences in the absence of any external stimuli, and paranoid delusions, representing fixed false beliefs not amenable to change in the face of new evidence.

It has been suggested that treatment resistance in schizophrenia is associated with a primary non-dopaminergic mechanism possibly underpinned by glutamatergic changes in prefrontal cortex. Supporting this, striatal dopamine synthesis capacity was reported as unchanged in resistant compared to responsive patients, while glutamate levels in anterior cingulate cortex (ACC) were increased (Demjaha et al., 2014). Recently, we have shown that fMRI BOLD-related RPE signalling in striatum is reduced in treatment responsive patients but intact in resistant patients, in the absence of any significant differences in their performance during a reinforcement learning task with an additional emotional bias parameter (Vanes et al., 2018). Whilst we did not measure dopamine function explicitly, the treatment responsive patients demonstrated impaired learning behaviour associated with a dysfunctional change in RPE signalling – associated with dopaminergic function. The RPE signal was also accentuated by the emotional bias (bias towards choosing the ‘happy face’ over ‘angry face’ even when there was less evidence that the happy face would be rewarded) in responsive patients. In contrast, the impairment in learning performance in the treatment resistant patients was associated not by the RPE signalling itself, but by dysfunctional interaction of the emotional bias on RPE (positive relationship between emotional bias score and RPE signal in thalamus). This showed that responsive and resistant patients had deficits in learning performance but through putatively different pathways. Thus, we propose a two-stage mechanistic theory of treatment-resistant schizophrenia whereby RPE signalling (based on dopaminergic function) is largely intact (and is only marginally improved by antipsychotic medication), but symptoms persist (in treatment resistant patients) as a consequence of a failure of cognitive control over the primary dopaminergic dysfunction in the striatum and sensory cortices. Here we investigate the mechanisms of cognitive control within a perceptual reward-learning paradigm and test the hypothesis that treatment-resistant patients will display impaired cognitive control - evidenced by reduced effective connectivity from ACC to sensory and reward regions - compared to responsive patients.

## 2 Methods and materials

A full description of the participants, reward learning task and the fMRI scanning parameters are available in Vanes et al. (2018).

### 2.1 Participants

Data from 21 responsive and 20 treatment-resistant medicated patients with a diagnosis of schizophrenia showing differential RPE signalling (Vanes et al., 2018) and twenty-four healthy controls (HC) were included in the analysis. Treatment resistance was determined based on persistent psychotic symptoms (score of 4 or more on at least two positive symptom items from the Positive and Negative Syndrome Scale (PANSS)), no clinical improvement from at least two prior antipsychotic drug trials lasting 4–6 weeks in duration and an illness duration of at least 5 years with no good period of social-occupational functioning. These criteria were assessed by reviewing patient medical records and occupational status (self-report). Treatment responsive patients were determined to be in symptomatic remission based on having scores of 3 or less on all items of the PANSS (Conley and Kelly, 2001) that were stable for at least 6 months and being prescribed a stable dose of antipsychotic medication for the 6 months prior to the study (Andreasen et al., 2005). Groups were matched for age, sex and socio-economic status, and the two patient groups were matched for age of illness onset, illness duration, medication dose (CPZ equivalent) and smoking status (please see Table 1 in (Vanes et al., 2018)). Please see supplementary methods for additional details including exclusion criteria. The London Camberwell St Giles Research and Ethics Committee provided ethical approval for the study beforehand and all participants provided written informed consent prior to participation.

### 2.2 Clinical rating scales and questionnaires

Clinical symptoms were assessed using the Positive and Negative Symptom Scale (PANSS) administered by research assistants following training (Kay et al., 1987). Aberrant salience was assessed using the Aberrant Salience Inventory (ASI) in all participants (Cicero et al., 2010). This is a 29-item self-report questionnaire suitable for clinical and non-clinical populations.

### 2.3 Reward learning task

**Fig. 1:**
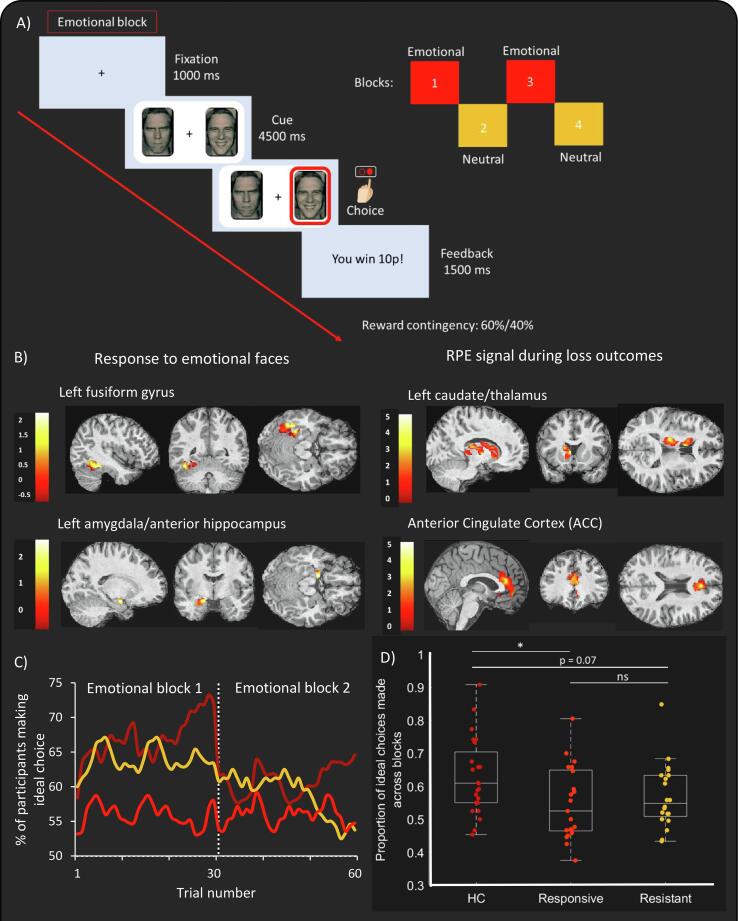
Overview of task. (A) Reinforcement learning task where participants had to maximise their monetary rewards by learning which face was associated with a 60% chance of being rewarded. (B) Average BOLD-related signal across participants in response to emotional faces (emotional - neutral face contrast) and RPE loss outcomes. These 4 regions were masked and extracted to form the network for DCM connectivity analysis. (C) Percentage of participants making ideal choices over time during the two emotional blocks (30 trials/block). This shows learning behaviour between groups. (D) Proportion of ideal choices made across emotional and neutral blocks by healthy controls (HC), treatment-responsive and treatment-resistant patients (white lines show significant differences between groups, * = p < 0.05, ns = non-significant).

Whilst undergoing fMRI scanning, participants were asked to choose one of two simultaneously presented faces and learn to identify which face was associated with a higher probability of reward over a series of 30 trials (for each block). There were four blocks in total: two ‘emotional’ blocks where participants chose between angry and happy facial expressions, and two ‘neutral’ blocks where participants chose between two neutral faces of different identities (please see Fig. 1A) (Evans et al., 2011). In each block, one face was associated with a higher probability of reward (60% vs. 40% contingency) where every correct choice (referred to as ‘wins’) was rewarded with 10p and every incorrect choice (referred to as ‘losses’) resulted in reward omission. Please refer to Vanes et al. (2018) for details of fMRI set up and Fig. 1A for timings (Vanes et al., 2018).

### 2.4 Imaging data analysis

The previously published fMRI data was analysed in FSL (Jenkinson et al., 2012). For the purposes of Dynamic Causal Modelling, fMRI preprocessing and analysis was replicated in Statistical Parametric Mapping, version 12 (SPM12, available at http://www.fil.ion.ucl.ac.uk/spm/software/spm12, Wellcome Department of Cognitive Neurology, London, England). First, the structural and functional images were skull-stripped and manually reoriented so that the origin was reset over the anterior commissure. Next, the functional images were realigned to correct for the effects of head motion, co-registered to the structural images and normalised to Montreal Neurological Institute (MNI) space. Finally, a temporal high pass filter of 100 s was applied, and the data were spatially smoothed using a 6 mm FWHM Gaussian kernel.

The general linear model was used to analyse the fMRI data. For the first-level analysis, there were 8 (unmodulated) regressors that modelled the conditions of the task (face presentation, decision, feedback from ‘win’ trials and feedback from ‘loss’ trials) separately for the emotional and neutral blocks. Additionally, the feedback phases of the task were parametrically modulated with trial-by-trial RPE values which added 4 (modulated) regressors to the model (i.e. RPE win (emotional), RPE win (neutral), RPE loss (emotional), RPE loss (neutral)), giving a total of 12 regressors. The RPE values were estimated using a ‘double update’ reinforcement learning model (Schlagenhauf et al., 2014) which uses the same Q-learning algorithm as the standard Rescorla-Wagner model but with the addition that the Q values (or expected outcome) for both the chosen and unchosen face are updated on every trial. For more details of this reinforcement learning model, please refer to (Vanes et al., 2018, Schlagenhauf et al., 2014). Each regressor was modelled with a delta function (duration set to zero) and was convolved with a canonical haemodynamic response function (hrf) and its temporal derivative. Six standard motion parameters and an additional subject-specific motion artifact confound matrix were added as regressors of no interest (please see supplementary methods for details of motion correction). To check the reproducibility of the findings, group-level mixed effects analyses were performed. Whole-brain activation patterns in response to ‘RPE win’ and ‘RPE loss’ were qualitatively replicated from the previous FSL analysis (e.g. in prefrontal cortices, frontal cortices, parietal cortices, visual cortices and cerebellum).

### 2.5 Behavioural analysis

The proportion of ideal choices made across the task was computed (excluding missing trials). Briefly, the participant’s choice was labelled as ‘ideal’ when their expected reward (Q_1_(t) estimated using the double-update reinforcement learning model) for the chosen face was greater than that of the unchosen face (see (Vanes et al., 2018) for details). Therefore, the ideal option can be thought of as how well the participant estimates and translates their value representation into their choice action. Since reward contingencies were similar (60%/40%), the task is difficult and so we expected the percentage of participants making ideal choices to increase gradually over trials as an index of learning. A one-way ANOVA with post-hoc t-tests (with Bonferroni correction) was also used to compare the proportion of ideal choices made across blocks between groups.

### 2.6 Dynamic causal modelling

Deterministic Dynamic Causal Modelling (DCM) was applied to examine effective connectivity within a cortico-striatal-limbic network comprising of four regions of interest (ROIs) activated during the reward learning task (Fig. 1B). These regions were carefully chosen based on previous literature showing the important role of cognitive control and cortico-striatal dysfunction in schizophrenia (Minzenberg et al., 2009, Kerns et al., 2004, Brown and Braver, 2005). First, a striatal region extending over the caudate and thalamus was chosen that was previously shown to be differentially activated between HC, responsive and resistant groups during RPE ‘loss’ trials (using a contrast that averaged across emotional and neutral conditions) (Vanes et al., 2018). A second region encompassing the anterior cingulate cortex (ACC) and part of the middle cingulate cortex was also activated during this contrast. These reward-related regions activate in response to RPEs where the ACC is involved in both reward prediction and cognitive control (Kerns et al., 2004, Brown and Braver, 2005). The fusiform gyrus and amygdala/anterior hippocampus were then chosen as sensory processing regions responding to visual and emotional information of ‘face’ stimuli (using an emotional – neutral faces contrast). These ROIs formed a network of interacting brain regions supporting cognitive control and reward learning. The time series from the peak functional activation was extracted from these ROIs for analysis (see Supplementary materials for details). Effective connectivity between these regions (caudate, ACC, fusiform and amygdala) was then assessed using DCM to investigate how this network supports decision-making and learning in HC and is impaired in treatment- responsive and resistant patients.

The DCM was specified using *a priori* knowledge of sensory processing and reward learning from the literature (visualised in Fig. S1). Two driving inputs were defined where ‘face’ cues were set to drive fusiform and amygdala, while regressors relating to reward feedback and RPE (during both emotional and neutral blocks, and win and loss trials) were set to drive the caudate and ACC (C matrix). Forward and backward fixed connections were considered between all regions except between amygdala and fusiform, and self-inhibitory connections were set for each region (A matrix). Task-related regressors were then set to modulate specific endogenous connections (B matrix); emotional face cues modulated forward connections out from the sensory regions (fusiform and amygdala) whereas the reward feedback and RPE regressors were set to modulate connections out from the RPE-related regions (caudate and ACC) as well as the connections between them. Model inversion was performed using DCM12.5 within SPM12 to calculate parameter estimates (connection strengths) for each participant. In particular, we tested which fixed connections in this brain network were present during the task and were specifically interested in top-down connections (from ACC) associated with cognitive control function.

### 2.7 Parametric Empirical Bayes (PEB)

The DCM was brought forward to a second-level Parametric Empirical Bayes (PEB) analysis where differences in effective connectivity between the two patient groups and healthy controls were tested (Zeidman et al., 2019) (see Supplementary Methods for details). We compared each patient group to the control group using two PEB models (responsive > HC and resistant > HC), given that patient labels are known, with the hypothesis that each patient group has a different underlying mechanism. A PEB model comparing responsive > resistant patients was then tested to directly compare the patient groups and confirm the previous PEB effects that were in relation to the HC group. Patient groups were compared separately using PEB as it is recommended that second level design matrices be somewhat constrained (Zeidman et al., 2019), however a complementary ‘omnibus’ model that included all three groups in one PEB model is also included. This allowed us to examine 1) the average effect across groups, 2) the additive effect of being a patient (both patient groups together) and 3) the additive effect of being treatment resistant.

Using the same PEB framework, top-down connectivity from the ACC to the perceptual-reward network was examined with respect to positive and negative symptoms, and aberrant salience (parametric covariates) (Cicero et al., 2010). For each group separately (HC, responsive, resistant), a PEB model was constructed modelling the group mean (constant term) and the mean-centred covariate of interest (e.g. positive symptoms) as regressors. This enabled us to test whether disrupted connectivity relates to symptoms and salience in each patient group. Due to our hypothesis of impaired cognitive control in treatment-resistant patients, PEB models for symptoms, salience and glutamate were examined in ‘top-down’ connections from ACC to fusiform, amygdala and caudate. Finally, PEB models were constructed for each group to relate top-down connectivity to ACC glutamate levels. This allowed us to test whether measures of glutamate within the ACC were related to the deployment of ACC connections, the most prominent candidate for a non-dopaminergic mechanism of treatment resistance. To formally compare whether the relationship between top-down connectivity and glutamate were different between groups, PEB models were constructed to confirm a group × glutamate interaction (see Supplementary Methods for details). The expected value (Ep) and associated posterior probability (Pp) are reported for each connection considered ‘significant’ (parameters with Pp values > 0.95).

### 2.8 MR spectroscopy

Glutamate concentrations were acquired from the ACC using a standard GE PROBE (proton brain examination) sequence to collect 1H-MRS spectra (Point Resolved Spectroscopy (PRESS) (see Supplementary materials for details). Spectroscopy data were analysed using LCModel version 6.3 (http://s-provencher.com/lcmodel.shtml) (Provencher, 2001) using a standard basis set of 16 metabolites (including glutamate). Metabolite concentration estimates were expressed as a ratio to total creatine (creatine + phosphocreatine). Glutamate was chosen as the appropriate metabolite to report (instead of glutamine (Gln) or Glx) because accurate quantification of Gln was not available at 3 T field strength.

### 2.9 Data availability

DCM/PEB code and data will be available on github. Imaging data will be stored on NeuroVault. All procedures contributing to this work comply with the ethical standards of the relevant national and institutional committees on human experimentation and with the Helsinki Declaration of 1975, as revised in 2008.

## 3 Results

### 3.1 Behaviour

Learning behaviour showed that the number of participants making ideal choices increased over 30 trials within each block (Fig. 1C). The proportion of ideal choices made across all blocks was significantly different between groups (*F*(2,62) = 3.54, *p* = 0.035) with healthy controls making significantly more ideal choices (*M* = 0.63, *S.D* = 0.13) compared to responsive (*M* = 0.55, *S.D* = 0.13, *p* = 0.013) and marginally more compared to resistant patients (*M* = 0.57, *S.D* = 0.13, *p* = 0.071) (Fig. 1D).

### 3.2 DCM network connectivity

**Fig. 2:**
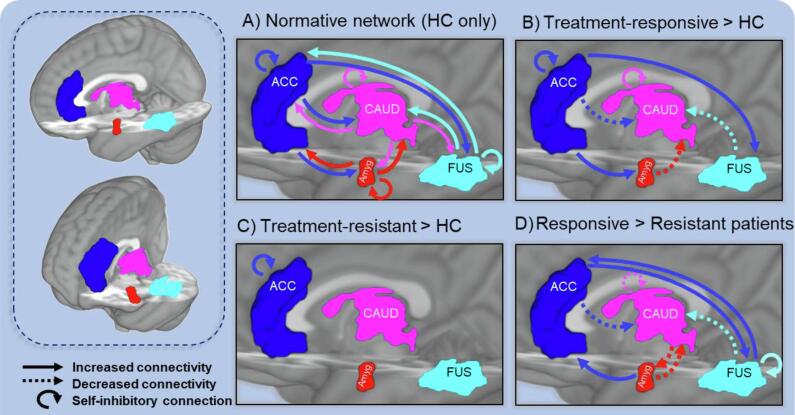
Network of interacting brain regions supporting reinforcement learning. A) displays significant connections in healthy controls showing all connections within the network are needed to perform this fMRI task. In comparison to the healthy controls (HC), B) shows connections between brain regions that are significantly altered in treatment-responsive patients and C) in treatment-resistant patients. In particular, top-down connectivity (from ACC to amygdala and fusiform) is increased in treatment-responsive but absent in treatment-resistant patients. Finally D) shows a complementary analysis directly comparing patient groups (connections that are significantly different in treatment-responsive compared to treatment-resistant patients). ACC = anterior cingulate cortex, CAUD = caudate, Amyg = amygdala, FUS = fusiform gyrus.

A normative model of effective connectivity was identified from HC participants (Fig. 2A). All specified endogenous connections were found to be significant excitatory connections or significant self-inhibitory connections (Pp > 0.95). During the task, face cues are processed in the sensory regions (fusiform and amygdala) before being processed by the caudate and ACC. Here, information about the stimulus (e.g. its reward value, salience, emotion) is integrated and reward predictions are formed. Relevant to the hypotheses, connectivity from fusiform to ACC was also positively modulated (or up regulated) by emotional face cues during the task (see Table. S2 for a complete list of significant modulators of connections in the normative model and Fig S2 for visualisation). During reward feedback, effective connectivity from ACC down to fusiform, amygdala and caudate was observed, consistent with top-down control of these regions. This is likely to represent the updating of reward predictions for future trials.

Network connectivity in responsive patients was tested relative to HC (Fig. 2B). Significantly increased top-down control of the fusiform and amygdala by the ACC was observed. In turn, there was reduced effective connectivity from ACC and sensory regions into the caudate. There was also increased self-inhibitory connectivity within the ACC and caudate indicating these regions ‘shut down’ more rapidly than in HCs. In contrast, the resistant group showed unaltered connectivity in this network compared to HC participants, except the inhibitory connectivity within the ACC was significantly increased (Fig. 2C). This suggests the network connectivity responsible for supporting RPE responses is intact in resistant patients, and that impaired sensory processing, RPE responses and dopaminergic drive may not be the core mechanisms underlying treatment resistant schizophrenia. Direct comparison of responsive vs. resistant patients showed that responsive patients had increased connectivity from ACC to sensory regions and reduced connectivity from the ACC and sensory regions to the striatum compared to resistant patients (Fig. 2D). This finding confirmed that altered connectivity in this network is driven by responsive patients and limited to the ACC in resistant patients (full list of connections presented in Fig. S3). The omnibus PEB model including the additive effect of being in the patient group and the additive effect of being treatment resistant revealed comparable effects to those reported from individual model comparisons (Table S1). No task-related modulators were significantly different in each group (Table. S2).

### 3.3 Connections relating to symptoms and salience

**Fig. 3:**
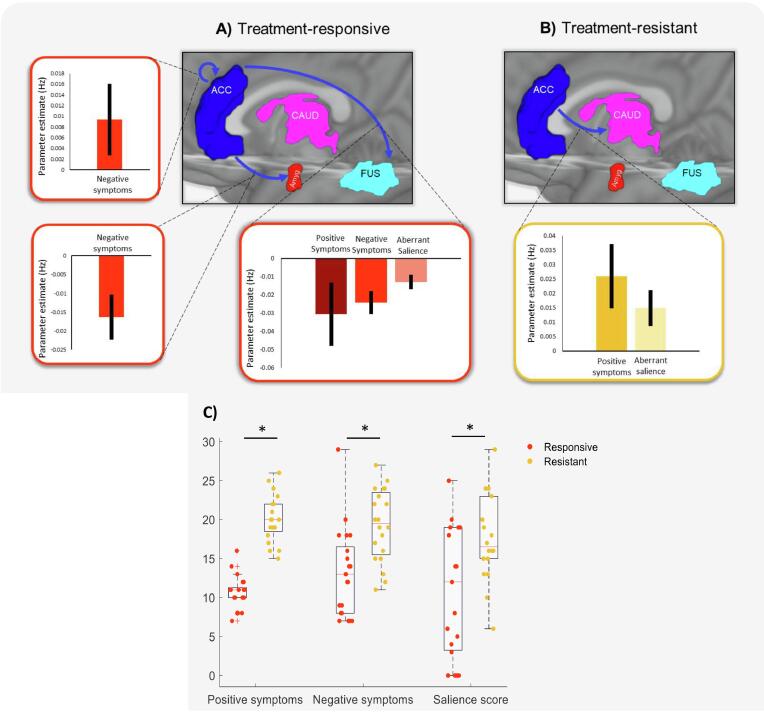
Top-down connectivity related to symptoms and salience. Top-down connections (from ACC) showing significant relationships with positive symptoms, negative symptoms and aberrant salience scores using Parametric Empirical Bayes (PEB) for (A) responsive and (B) resistant patients (Pp > 0.95). Bar charts show the expected values (Ep) with 90% Bayesian confidence intervals. (C) Box plots showing resistant patients have significantly higher positive symptoms, negative symptoms and aberrant salience scores compared to responsive patients (*t*-test, * = p < 0.05).

The clinical and behavioural consequences of this altered network were confirmed: the key top-down connections from the ACC to the reward network were correlated with both the positive and negative symptom (PANSS) scores and measures of aberrant salience scores in each group (Kay et al., 1987, Cicero et al., 2010). Resistant patients had significantly higher positive symptoms (hallucinations, delusions, disorganised thinking) (*M* = 20.5, *S.D.* = 3.1, *χ^2^* = 12.1, *p* < 0.001) and negative symptoms (amotivation, anhedonia, social withdrawal) (*M* = 19.5, *S.D*. = 4.6, *χ^2^* = 4.08, *p* < 0.001) compared to responsive patients (*M* = 10.7, *S.D*. = 2.1, *M* = 13.1, *S.D*. = 4.6, respectively) (Fig. 3B). Resistant patients also had significantly higher aberrant salience scores (*M* = 17.67, *S.D*. = 5.67) than responsive patients (*M* = 11.11, *S.D*. = 8.82, *p* = 0.023) and HC participants (*M* = 6.87, *S.D*. = 6.89, *p* < 0.001) (Fig. 3C). There was no difference in aberrant salience scores between HC and responsive participants (*p* = 0.18).

In the responsive group, reduced negative symptom severity was related to increased connectivity from ACC to sensory regions (fusiform and amygdala) and reduced ACC self-inhibition (Fig. 3A). Positive symptom severity and aberrant salience scores were also inversely related to the connection from ACC to fusiform (Effect size = −0.03 Hz, Pp = 1), demonstrating the beneficial effects of enhanced ACC control (relative to HCs) in those who respond to medication. In the resistant group however, positive and negative symptoms were not related to effective connectivity from ACC to sensory regions. This further suggests that top-down control of sensory information appears unaltered in resistant patients. However, effective connectivity from ACC to caudate was positively related to both positive symptoms (Effect size = 0.026 Hz, Pp = 1) and aberrant salience (Effect size = 0.015 Hz, Pp = 1) in resistant patients (Fig. 3B). Scatter plots of these relationships can be found in Fig. S4 for display purposes only.

### 3.4 Glutamate

**Fig. 4:**
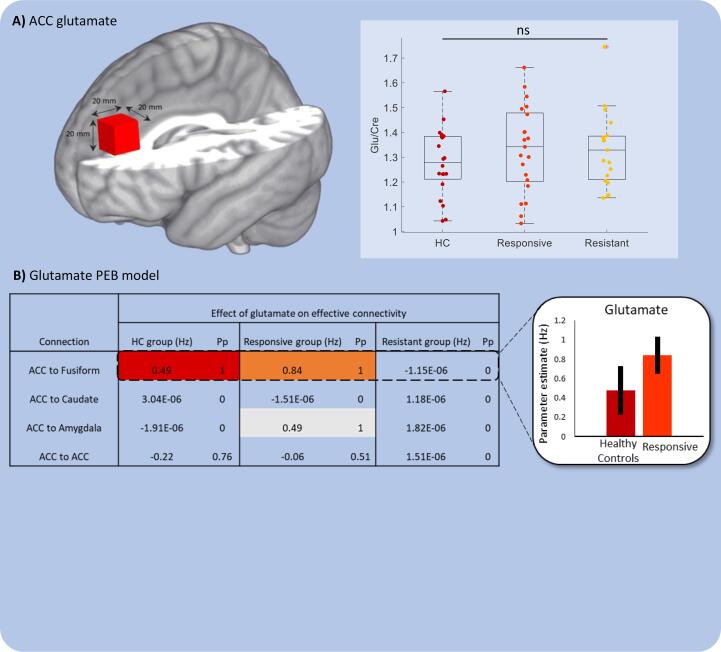
Top-down connectivity and glutamate. (A) Box plot showing no significant difference in glutamate levels (measured from ACC and referenced to creatine levels) between all three groups. (B) Top-down connections (from ACC) relating to glutamate in each individual group PEB (highlighted cells show significant effects (Pp > 0.95)). Top-down effective connectivity in resistant patients is not related to glutamate (Pp’s = 0). This difference was confirmed in a PEB model showing a significant negative group × glutamate interaction for resistant > HC and resistant > responsive. Bar chart shows the expected values (Ep) with 90% Bayesian confidence intervals for the ACC to fusiform connection.

Finally, glutamate levels in the ACC were examined against the top-down connections from the DCMs. This allowed us to determine whether cognitive control mechanisms were associated with glutamatergic signalling in resistant patients. Glutamate levels (referenced to total creatine) did not significantly differ between HC participants (*M* = 1.28, *S.D*. = 0.14), responsive patients (*M* = 1.33, *S.D*. = 0.18) and resistant patients (*M* = 1.33, *S.D*. = 0.15, *F*(2,59) = 0.72, *p* = 0.49) (Fig. 4A). Removing one outlier from the resistant group did not change this (*F*(2,58 = 0.65, *p* = 0.53). There were no significant differences between groups in the other metabolites; Glx, NAA, Choline, Myo-inositol (See Table S3).

Effective connectivity from ACC to fusiform was positively related to glutamate levels in both the HC (Effect size = 0.48 Hz, Pp = 1) and responsive patients (Effect size = 0.84 Hz, Pp = 1) (Fig. 4B). No other connections were related to glutamate in the HC group but connectivity from ACC to amygdala was positively related to glutamate in responsive patients (Effect size = 0.48 Hz, Pp = 1). However, in the resistant group, none of the connections were related to glutamate (all Pp’s = 0) (Fig. 4C); suggesting that glutamate may not be utilised effectively to support cognitive control in this resistant group.

Between-group comparisons demonstrated a significant negative group × glutamate interaction on the connection from ACC to fusiform for resistant > HC (Effect size = −0.049 Hz, Pp = 1) and resistant > responsive (Effect size = −0.061 Hz, Pp = 1) but not for responsive > HC. There was also a significant group × glutamate interaction in the resistant > responsive for the ACC to amygdala connection (Effect size = −0.032 Hz, Pp = 0.95) and ACC to caudate connection (Effect size = 0.082 Hz, Pp = 1) and these connections were borderline significant for the resistant > HC comparison too (see Table S3). Finally, there was a negative interaction between glutamate and responsive > HC group for the ACC to caudate connection (Effect size = −0.058 Hz, Pp = 1). These findings confirm that the relationship between ACC glutamate levels and top-down connectivity is altered in the treatment resistant patients compared to the other two groups.

## 4 Discussion

Our findings support the notion that treatment resistance represents a subtype of schizophrenia with a distinct underlying neurobiological mechanism. Treatment responsive patients demonstrated increased connectivity from ACC to sensory regions and decreased connectivity from all regions into the striatum. We interpret this pattern of connectivity as enhanced top-down control of sensory input to the striatum and a ‘compensatory’ mechanism unique to treatment responsive patients. This suggests a key mechanism that is needed to supplement antipsychotic blockade of D2 receptors in the core reward circuitry (although dopamine function was not explicitly measured in this study). In treatment-resistant patients, no such ‘compensatory’ control exists. Indeed, connectivity within this reward network was very similar to healthy controls and the RPE response in striatum (as reported previously (Vanes et al., 2018)), associated with dopaminergic signalling, was intact. Instead, self-inhibitory connectivity within the ACC was increased in resistant patients, and ACC-striatal ‘hyper’-drive was associated with both increased psychotic symptoms and aberrant salience. This suggests that ACC function may be inefficient in resistant patients and that symptoms may persist if top-down regulation of the reward network is impaired – a mechanism that is not targeted by current antipsychotic medication (Lowe et al., 2018). The specificity of this finding would be best addressed using a prospective longitudinal design, with larger numbers of patients, to test the potential of these effective connectivity measures to predict treatment resistance.

Effective reward learning requires cognitive control processes responsible for integrating sensory information, allowing effective contextualisation of (non) salient distractors and adjusting behaviour appropriately (Newman et al., 2015). This is associated with activation of the prefrontal cortex, including the ACC. Thus, impaired top-down control by the ACC may permit dysfunctional evaluation of task-relevant information serving to update the caudate with inaccurate reward predictions. This was associated with positive psychotic symptoms and aberrant salience. This finding is supported by evidence that prefrontal-striatal functional connectivity is important for determining treatment response in antipsychotic-naïve patients with psychosis. For example, Cadena et al., 2018, Cadena et al., 2019) reported increased ACC functional activity and functional connectivity between ACC and putamen from baseline to follow-up was associated with better treatment response (decrease in symptoms) following a 6-week course of antipsychotic medication (risperidone) (Cadena et al., 2018, Cadena et al., 2019). Sarpal et al. (2016) also reported that resting-state striatal functional connectivity, including with ACC, was predictive of treatment response following a trial of second generation antipsychotic medication in first episode psychosis (Sarpal et al., 2015). Here, we demonstrate that altered top-down connectivity from ACC to the extended reward network (both sensory regions and striatum) differentiates treatment responsive from treatment resistant patients with chronic schizophrenia and this relates to clinical symptoms and glutamate function.

Some earlier data have demonstrated significantly increased ACC glutamate levels in resistant compared to responsive patients (Demjaha et al., 2014, Merritt et al., 2016, Mouchlianitis et al., 2016) although findings are inconsistent (Kumar et al., 2020). This finding was not replicated in the current sample. In addition to methodological heterogeneity across published studies, an inherent limitation of MRS at 3 T is that it is a relatively crude index of glutamate concentration. 1H-MRS does not measure NMDARs specifically but total glutamate within a voxel. This includes mGluRs, which are also implicated in the psychopathology of schizophrenia (Nicoletti et al., 2019). Given the heterogeneity in clinical profiles, even within treatment-resistant and treatment-responsive subtypes, the specific nature of glutamatergic dysfunction that might underlie treatment-resistance is yet to be established. The current study showed that ACC glutamate levels were unrelated to top-down connectivity in resistant patients, contrary to HC and responsive patients. This highlights a key strength of the multi-modal approach adopted in the present study, allowing to identify differential glutamate-related connectivity effects between resistant and responsive patients, even in the absence of ACC glutamate differences. We suggest this is likely to represent dysfunctional glutamatergic signalling failing to support optimal cognitive control in treatment resistant patients. Indeed, treatment-resistant schizophrenia has been associated with more marked cognitive deficits (Frydecka et al., 2016) and both animal and human models of schizophrenia indicate NMDA receptor hypofunction (Moghaddam et al., 1997). Our observations support an alternative, non-dopaminergic mechanism for treatment-resistant schizophrenia. This finding is important for developing new drugs (e.g. glutamatergic targets) and guiding treatment strategies (e.g. prescribing clozapine earlier). Future research into modulating cognitive control mechanisms (Orlov et al., 2017) and glutamate function will be useful to confirm this putative pathology in treatment resistance.

## 5 Limitations

The present findings should be considered with respect to some limitations. First, the modest sample size (24 healthy, 21 responsive, 20 resistant). Recruiting patients with treatment-resistant schizophrenia is challenging and whilst we have tried to counteract this issue by using a generative modelling approach and a clearly-defined hypothesis, it should be noted that power is limited and that replication in a larger, independent sample is warranted. Second, all patients in the study were medicated at the time of the study, excluding clozapine. Although patient groups were matched for illness duration and medication dosage (CPZ equivalent), treatment with antipsychotics has been related to changes in dopamine synthesis capacity (Howes et al., 2011) and brain function including cortico-striatal functional connectivity (Sarpal et al., 2015). We are therefore unable to determine the effect of medication on the current findings. Future longitudinal studies comparing brain connectivity before and after treatment with antipsychotic medication in first episode patients with psychosis will be important for predicting treatment resistance. Third, fMRI signal was extracted from relatively broad regions of interest at low thresholds. The regions were chosen a priori based on previously published findings and we allowed for inter-individual variability in activation for the patient population. However, this limits the precision of connectivity patterns in this network; for example, connectivity to the amygdala and anterior hippocampus cannot be differentiated. Forth, we are unable to exclude the effects of glutamine contamination on the reported glutamate concentrations. It is estimated that the glutamate and glutamine peaks overlap < 30% in the range of 2.25–2.55 ppm with short TEs < 40 ms (Snyder and Wilman, 2010). It was therefore estimated that glutamine contributes < 10% to the glutamate signal in the current study (similar to (Mouchlianitis et al., 2016)) and future studies at higher field strengths (e.g. 7 T) would be useful to better quantify glutamate concentrations. Finally, the group-level findings are based on the PEB framework where DCM parameters are re-estimated using a prior (group membership). This allowed us to test where effective connectivity is different in this reward learning network, assuming each group is different. However, the current findings do not speak to being able to separate participants based on network connectivity alone. This is an aim for future studies where predefined connections can inform unsupervised models of schizophrenia.

## 6 Conclusion

We observe a distinction between responsive and resistant patients; that responsive patients display effective compensatory control of sensory precision during the task (increased top-down connectivity from ACC to sensory regions serving to reduce sensory input to the striatum) and an absence of this compensatory cognitive control mechanism in resistant patients. We suggest that this presents an alternative mechanism impacting learning and decision making in resistant patients with schizophrenia.

## CRediT authorship contribution statement

**Charlotte M. Horne:** Conceptualization, Methodology, Formal analysis, Writing - original draft, Writing - review & editing, Visualization, Project administration. **Lucy D. Vanes:** Investigation, Project administration, Writing - review & editing. **Tess Verneuil:** Project administration, Writing - review & editing. **Elias Mouchlianitis:** Investigation, Project administration, Writing - review & editing. **Timea Szentgyorgyi:** Investigation, Project administration. **Bruno Averbeck:** Resources, Methodology, Writing - review & editing. **Robert Leech:** Conceptualization, Methodology, Writing - review & editing, Visualization. **Rosalyn J. Moran:** Conceptualization, Methodology, Software, Writing - review & editing, Visualization, Supervision. **Sukhwinder S. Shergill:** Conceptualization, Resources, Writing - review & editing, Supervision, Funding acquisition, Project administration.
