# Supplementary material for: Cognitive control network connectivity differentially disrupted in treatment resistant schizophrenia
Source: Neuroimage Clin. 2021 Mar 17;30:102631. doi: 10.1016/j.nicl.2021.102631 (PMC8044714; doi:10.1016/j.nicl.2021.102631)
Supplement: Supplementary data 1 [file mmc1.docx]

**Supporting Information**

**Supplementary Methods**

*Participants*

Exclusion criteria for all participants were: current clozapine use, a history of neurological illness, a major physical illness, drug dependency in the previous 6 months prior to the study (assessed via self-report and medical notes) and contraindication to MRI. For healthy controls, participants were excluded if they reported a history of psychiatric illness or a fist-degree relative with a history of psychotic illness.

*Head motion quality control*

As well as the six standard motion parameters that are estimated by SPM, DVARS was used to calculate and identify volumes corrupted by motion (*1*). This was implemented using FSL Motion Outliers and detected larger ‘spikes’ in head movement for each participant. This motion artifact confound matrix was added along with the six standard motion parameters as regressors of no interest in the first-level model. As reported in Vanes et al (2018), the percentage of corrupted volumes was not significantly different between healthy control, treatment-responsive and treatment-resistant groups (F(2,60) = 0.166, p = 0.848) (*2*).

*MR Spectroscopy*

Glutamate concentrations were acquired using a standard GE PROBE (proton brain examination) sequence to collect 1H-MRS spectra (Point Resolved Spectroscopy (PRESS); T_R_ = 3000 ms, T_E_ = 30 ms, 96 averages). Before each scan, an auto-prescan was performed in order to optimise shimming and initial localiser scans and structural images were acquired in order to locate the ACC. Axial 2d T2-weighted fast spin echo scans and axial fast fluid-attenuated inversion recovery scans were acquired for this. A voxel measuring 20 x 20 x 20 mm was manually placed over the ACC in each participant by locating the centre of the voxel using the midline sagittal localiser slice and placing the voxel 13 mm above the genu of the corpus callosum, perpendicular to the AC-PC line. In line with hypotheses, glutamate (Glu/Cr) was the primary metabolite of interest, however four other metabolites (N-acetyl aspartate (NAA), Myo-inositol (ml), Choline (GPC+PCh) and Glx (glutamate + glutamine) were also explored. Only metabolite concentration estimates with a Cramer-Rao lower bound (CRLB) <20%, a signal-to-noise ratio > 10 and a linewidth of FWHM < 0.1 ppm were included in analyses.

*Dynamic Causal Modelling*

Dynamic Causal Modelling (DCM) for fMRI is a computational framework that models the hidden neural states underlying observed BOLD signal interactions to infer effective connectivity between brain regions (*3*). This allows *causal* interactions between brain regions to be modelled compared to more elementary functional connectivity approaches. The framework uses a bilinear model to estimate the change in neural state as a function of three types of coupling parameters; endogenous (fixed) connections between brain regions, task-related modulatory effects on these regions and experimental driving inputs (*3*). Haemodynamic parameters are estimated with the neuronal model to map the hidden neural state to the observed data (*4*). The time series from each ROI (ACC, caudate, amygdala, fusiform gyrus) were manually extracted for DCM analysis from each participant’s first-level SPM analysis. The peak functional activation for each region was located by the first author by applying a low threshold (p < 0.2 uncorrected) and the eigenvariate was extracted using a 6mm sphere. For the caudate and ACC regions, the time series were extracted during RPE loss trials (using a contrast that averaged across emotional and neutral blocks) whereas the fusiform and amygdala regions were extracted from when participants were presented with the facial expressions using an ‘emotion’ contrast (emotional faces – neutral faces). For simplicity, the caudate, amygdala and fusiform were extracted from the left hemisphere only where the group-level effect was stronger. One participant’s data was excluded from the study because there was no above threshold signal to extract for DCM analyses, leaving 65 participants (24 HC, 21 responsive, 20 resistant).

*PEB analysis*

The DCM model was brought forward to a second-level Parametric Empirical Bayes (PEB) analysis (*5*) (or group-level analysis) to examine where the mean endogenous connections were expressed in each group, between groups (HC, responsive, resistant) and in relation to a-priori covariates (e.g. ACC glutamate variability). This group-level analysis comprises a Bayesian estimate of group covariates (similar to a classical general linear model). First, the PEB analysis collates the estimated DCM parameters from each participant including the expected values of the parameters, their associated covariances and approximate likelihoods. Then, the individual DCM connectivity parameters were re-estimated using group membership as a prior on connectivity differences. Then, Bayesian model reduction is performed where a search is conducted over nested DCMs (i.e. combinations of all connections that differ according to group assignment) and prunes away parameters that, on average, don’t contribute to the group model evidence by comparing them against the fully connected models. Bayesian model averaging is then performed showing the group-level connection strengths averaged over the PEB models identified in the search. Two types of PEB models were constructed; the first type was used to identify differences in endogenous connections between two groups (e.g. resistant vs. HC) by specifying a design matrix with a group mean plus a regressor modelling group membership (1, -1). This means PEB re-estimation was performed using group membership as the prior. The second type of PEB model was used to identify group-level effects associated with a covariate (e.g. positive symptoms) by constructing separate PEB models for each group (HC, responsive, resistant) and modelling the group mean plus covariate of interest in the design matrix.

For each PEB model, a pipeline was applied: 1) the covariate of interest (e.g. group membership or positive symptoms) was mean-centred, 2) the PEB model was estimated for each field (A, B, C) separately using **spm_dcm_peb** where the DCMs are re-estimated, 3) Bayesian model reduction and averaging was performed using **spm_dcm_bmc_peb** (showing *if* there is a significant effect of group) and 4) using **spm_dcm_peb_bmc** (showing *where* the group effect is significant). For each PEB model, a design matrix was specified where the first regressor modelled the group mean (constant term) and the covariates of interest were added as the subsequent regressors.

The following PEB models were constructed:

1. The group mean for HC participants. This was to establish the normative model of network connectivity supporting reward learning (n =24).
2. Effect of patient group. Two separate PEB models to compare endogenous connectivity and modulatory inputs for the two patient groups compared to the HC participants (responsive > HC and resistant > HC). This allowed us to test which endogenous connections were specifically altered in each patient group. A PEB model comparing responsive > resistant groups was also run to compare endogenous connectivity between groups and confirm the effects in relation to the HC group (responsive = 21, resistant = 20). Parametric Empirical Bayes utilises a quasi-greedy search when testing for group effects from multiple possible parameter configurations. Thus, it is recommended that second level design matrices be somewhat constrained. That said, we also performed a complementary ‘omnibus’ model that included all three groups in one PEB model. This omnibus model allowed us to examine 1) the average effect across groups, 2) the additive effect of being a patient (both patient groups together) and 3) the additive effect of being treatment resistant. Due to our hypothesis of impaired cognitive control in treatment-resistant patients and the results of this group PEB, the following PEB models for symptoms, salience and glutamate were run for all connections but only the top-down connections (from ACC) were examined.
3. Effect of symptoms. Separate PEB models for each patient group investigating the effect of positive and negative symptoms (from the PANSS) on effective connectivity (responsive = 21, resistant = 20). This enabled us to test whether disrupted connectivity relates to symptoms (and salience below) in each patient group.
4. Effect of aberrant salience. Two separate PEB models investigating the effect of a behavioural measure of aberrant salience (ASI scores) on effective connectivity for responsive and resistant groups (n = 19, n = 18, respectively).
5. Effect of glutamate. Three separate PEB models investigating the effect of MRS glutamate (measured from the same region of the ACC) on effective connectivity for each group (HC = 20, responsive = 21, resistant = 19). Findings from these PEBs were examined in relation to top-down connections from ACC relating to the MRS voxel location and cognitive control. This allowed us to test whether measures of glutamate within the ACC were related to the deployment of ACC connections, the most prominent candidate for a non-dopaminergic mechanism of treatment resistance.
6. Group x glutamate interaction. Three separate PEB models were constructed to formally compare the group interaction with the parametric effect of glutamate (responsive > HC, resistant > HC and resistant > responsive). Each PEB modelled the mean effect and interaction effect (e.g. resistant glutamate values * 1, and HC glutamate values * -1 to form the single ‘interaction’ regressor where we searched for specific connections associated with this regressor).

The PEB framework uses Bayesian statistics and returns each parameter in terms of the expected value (Ep) (measured in Hz – given that connectivity in DCM is technically a rate constant) and associated posterior probability (Pp). Only parameters with Pp values > 0.95 were considered significant and reported in the text. The PEB framework was used because it uses the covariance of the expected values estimated in the first-level DCM to inform group level inference. This allows noisy data with uncertain parameter estimates to be down-weighted (*5*).

**Supplementary figures**

**
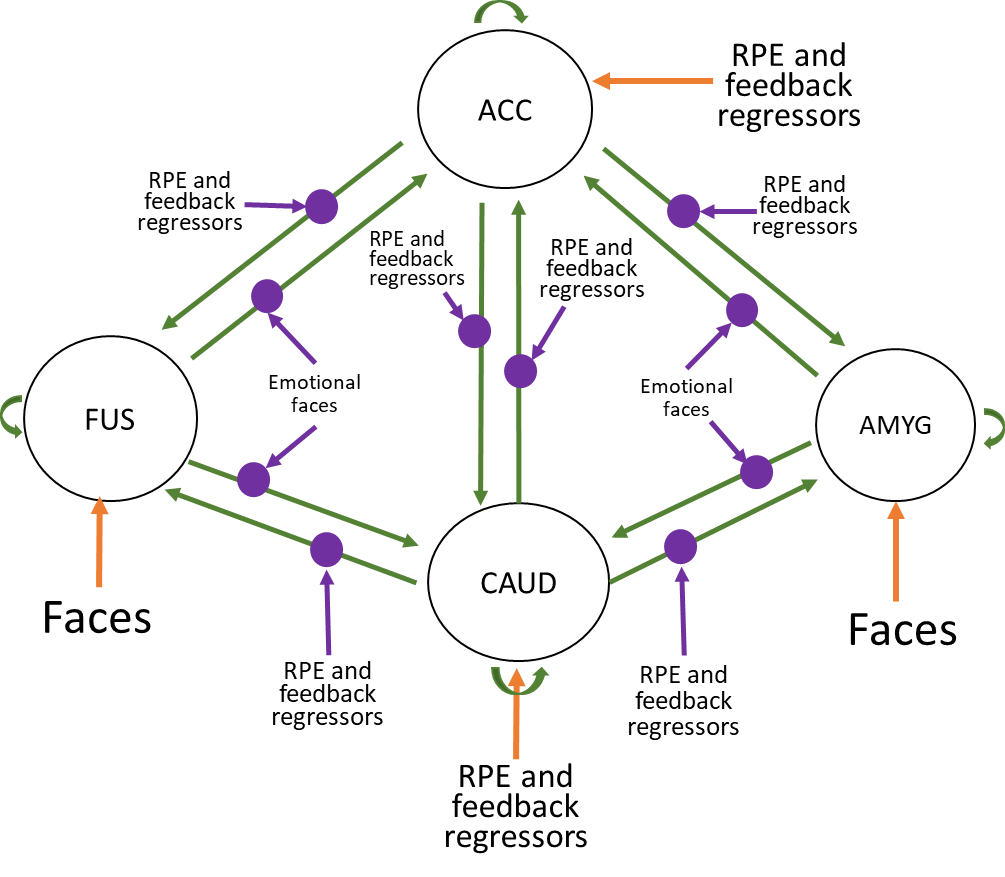
**


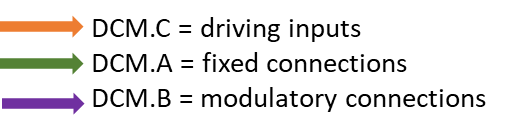


**Figure S1. Full DCM model set-up.** Fixed connections are specified between all regions except fusiform cortex (FUS) and amygdala (AMYG). All regions have inhibitory self-connections to prevent spurious activity arising from positive feedback loops. All face cues are set as driving inputs to sensory regions (fusiform cortex and amygdala) and emotional faces modulate the connections out of these two regions. Reward prediction error (RPE) and task regressors relating to reward feedback are set as driving inputs to RPE-related regions (caudate (CAUD) and anterior cingulate cortex (ACC)). They also modulate the connections out of these two regions and between them.

C) Selected significantly modulated connections

B) Significant endogenous connectivity


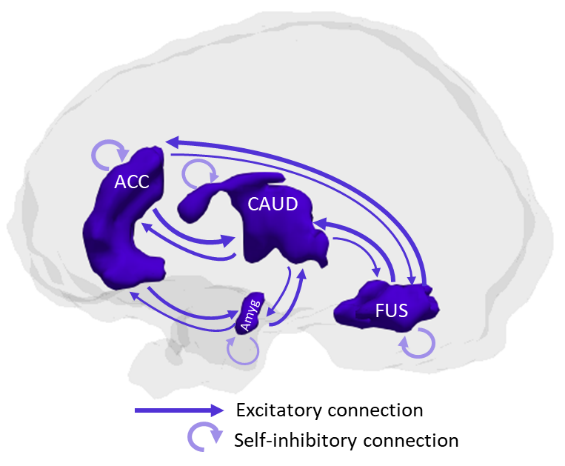

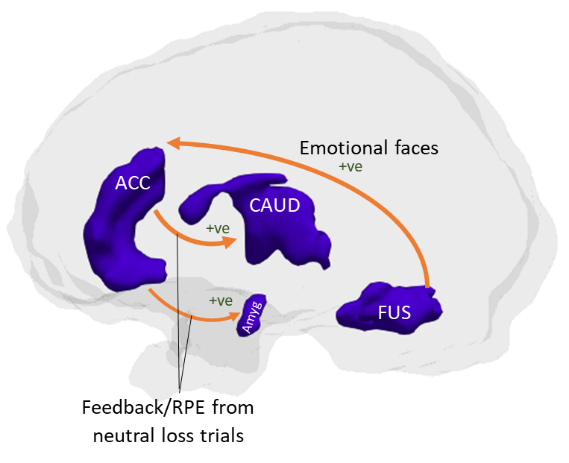


A) Driving inputs


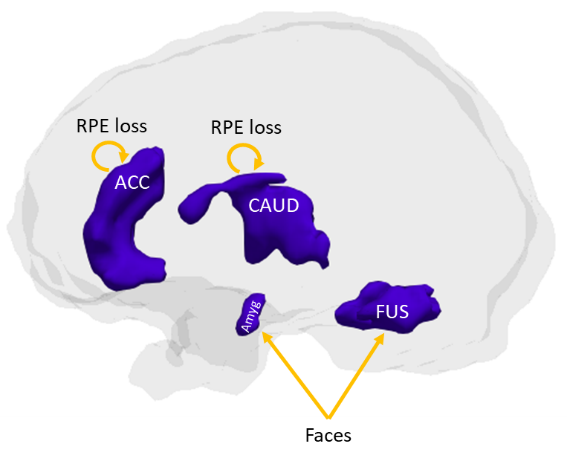


**Figure S2. Normative network function (healthy controls only).** The four regions in the network are overlaid onto a glass brain and (A) shows the driving inputs to these regions. (B) A PEB model run only in healthy controls (HC) reveals that all endogenous (fixed) connections between regions are significant and contribute to this network. (C) The same PEB model in HCs reveals that emotional faces and feedback/RPE regressors on loss trials (relevant to main GLM findings reported previously) are significant modulators of three connections. All other significant modulators are reported in Table S2 below.

**Table S1 Omnibus PEB model results.** Fixed connections showing significant connectivity (as indexed by Pp > 0.95) where all three groups are modelled in one PEB model. Results are comparable to PEB models comparing the groups separately. Fus = fusiform, caud = caudate, amyg = amygdala, ACC = anterior cingulate cortex.

**Table S2. All task-modulated ‘B’ connections.** Task-related regressors showing significant modulatory effects on connections (as indexed by the Posterior probability (Pp) > 0.95) where the parameter estimates show the size and direction of the effect (in bold). This is shown for PEBs run on HC only and Patients > HC as PEBs run for each patient group separately show no significant effects.

| Task-related modulators (B) | HC only | | Patients > HC | |
| --- | --- | --- | --- | --- |
|  | Parameter estimate (Hz) | Pp | Parameter estimate (Hz) | Pp |
| Emotional faces |  |  |  |  |
| Fus 🡪 Caud | 0.13 | 0.58 | 4.20E-06 | 0 |
| Fus 🡪 ACC | **0.61** | **1** | **-0.28** | **1** |
| Amyg 🡪 Caud | 3.28E-06 | 0 | 3.00E-07 | 0 |
| Amyg 🡪 ACC | 1.07E-01 | 0.54 | 1.89E-07 | 0 |
| Feedback emotional win |  |  |  |  |
| Caud 🡪 Fus | -4.58E-06 | 0 | 2.99E-06 | 0 |
| Caud 🡪 Amyg | **-0.22** | **1** | 1.35E-06 | 0 |
| Caud 🡪 ACC | -4.57E-06 | 0 | **-0.19** | **1** |
| ACC 🡪 Fus | -0.09 | 0.53 | -1.79E-06 | 0 |
| ACC 🡪 Caud | **-0.55** | **1** | **0.23** | **1** |
| ACC 🡪 Amyg | **-2.24E-01** | **1** | 0.052 | 0.50 |
| RPE emotional win |  |  |  |  |
| Caud 🡪 Fus | -1.26E-06 | 0 | 2.36E-06 | 0 |
| Caud 🡪 Amyg | 1.38E-06 | 0 | 2.73E-06 | 0 |
| Caud 🡪 ACC | 7.75E-06 | 0 | 4.83E-06 | 0 |
| ACC 🡪 Fus | 3.36E-06 | 0 | -2.94E-06 | 0 |
| ACC 🡪 Caud | -4.07E-06 | 0 | -4.36E-07 | 0 |
| ACC 🡪 Amyg | **2.06E-01** | **1** | -3.64E-06 | 0 |
| Feedback neutral win |  |  |  |  |
| Caud 🡪 Fus | -2.88E-06 | 0 | 1.43E-06 | 0 |
| Caud 🡪 Amyg | **-2.51E-01** | **1** | 3.91E-06 | 0 |
| Caud 🡪 ACC | **-3.20E-01** | **1** | 0.08 | 0.64 |
| ACC 🡪 Fus | 7.12E-06 | 0 | 4.61E-06 | 0 |
| ACC 🡪 Caud | **0.65** | **1** | **-0.31** | **1** |
| ACC 🡪 Amyg | **-2.34E-01** | **1** | -2.26E-06 | 0 |
| RPE neutral win |  |  |  |  |
| Caud 🡪 Fus | **-0.28** | **1** | **0.16** | **1** |
| Caud 🡪 Amyg | -4.66E-06 | 0 | 3.93E-06 | 0 |
| Caud 🡪 ACC | **-0.28** | **1** | **0.15** | **1** |
| ACC 🡪 Fus | -8.67E-02 | 0.51 | -7.93E-08 | 0 |
| ACC 🡪 Caud | 1.05E-01 | 0.54 | -0.06 | 0.55 |
| ACC 🡪 Amyg | -1.14E-01 | 0.58 | -1.96E-06 | 0 |
| Feedback emotional loss |  |  |  |  |
| Caud 🡪 Fus | -1.03E-06 | 0 | -3.19E-06 | 0 |
| Caud 🡪 Amyg | -3.65E-09 | 0 | -6.26E-06 | 0 |
| Caud 🡪 ACC | 1.61E-06 | 0 | -4.23E-07 | 0 |
| ACC 🡪 Fus | 2.74E-06 | 0 | -2.05E-06 | 0 |
| ACC 🡪 Caud | -7.83E-07 | 0 | -1.56E-06 | 0 |
| ACC 🡪 Amyg | 2.15E-06 | 0 | -1.07E-07 | 0 |
| RPE emotional loss |  |  |  |  |
| Caud 🡪 Fus | 3.83E-06 | 0 | -4.59E-06 | 0 |
| Caud 🡪 Amyg | -1.17E-06 | 0 | 1.74E-06 | 0 |
| Caud 🡪 ACC | 1.53E-06 | 0 | 1.06E-06 | 0 |
| ACC 🡪 Fus | -1.47E-06 | 0 | -2.68E-06 | 0 |
| ACC 🡪 Caud | -6.12E-06 | 0 | -1.52E-06 | 0 |
| ACC 🡪 Amyg | 1.24E-06 | 0 | -3.15E-06 | 0 |
| Feedback neutral loss |  |  |  |  |
| Caud 🡪 Fus | -4.96E-07 | 0 | 4.04E-06 | 0 |
| Caud 🡪 Amyg | 4.71E-06 | 0 | -6.28E-07 | 0 |
| Caud 🡪 ACC | 9.06E-07 | 0 | -1.13E-06 | 0 |
| ACC 🡪 Fus | 1.23E-01 | 0.58 | -0.09 | 0.64 |
| ACC 🡪 Caud | -2.69E-06 | 0 | 2.02E-06 | 0 |
| ACC 🡪 Amyg | **0.23** | **1** | **-0.17** | **1** |
| RPE neutral loss |  |  |  |  |
| Caud 🡪 Fus | -2.16E-06 | 0 | -1.29E-06 | 0 |
| Caud 🡪 Amyg | 1.13E-06 | 0 | 6.50E-07 | 0 |
| Caud 🡪 ACC | -5.95E-06 | 0 | -1.00E-06 | 0 |
| ACC 🡪 Fus | 0.13 | 0.58 | -0.06 | 0.54 |
| ACC 🡪 Caud | **0.37** | **1** | **-0.21** | **1** |
| ACC 🡪 Amyg | **0.37** | **1** | **-0.21** | **1** |


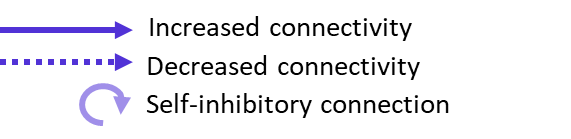

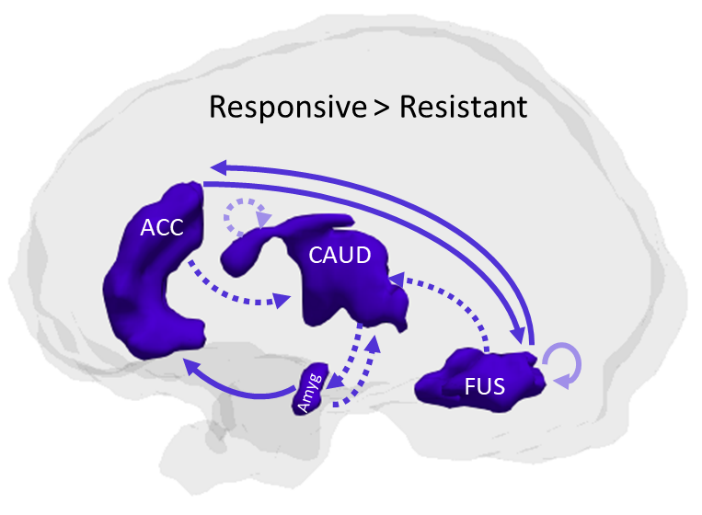

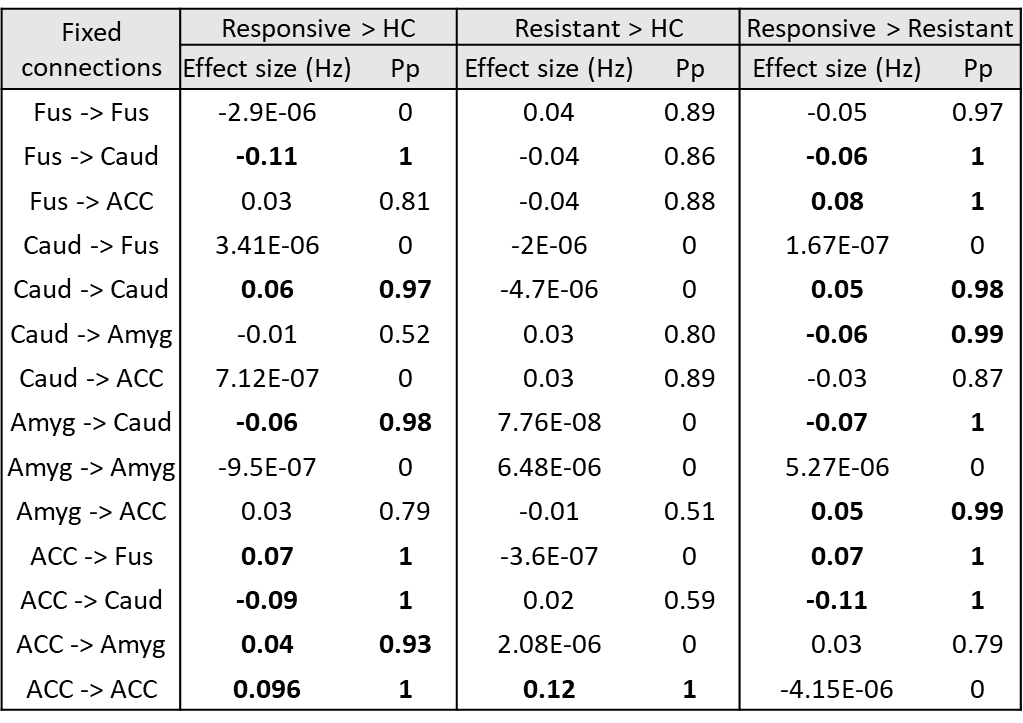


(A)

(B)

**Figure S3. Group PEB results directly comparing patient groups.** (A) shows all fixed connection parameter estimates and associated Posterior probabilities (Pp) for each group PEB model. Significant connections are shown in bold. (B) Significantly different connections displayed for responsive patients compared to resistant patients (Pp > 0.95).

**
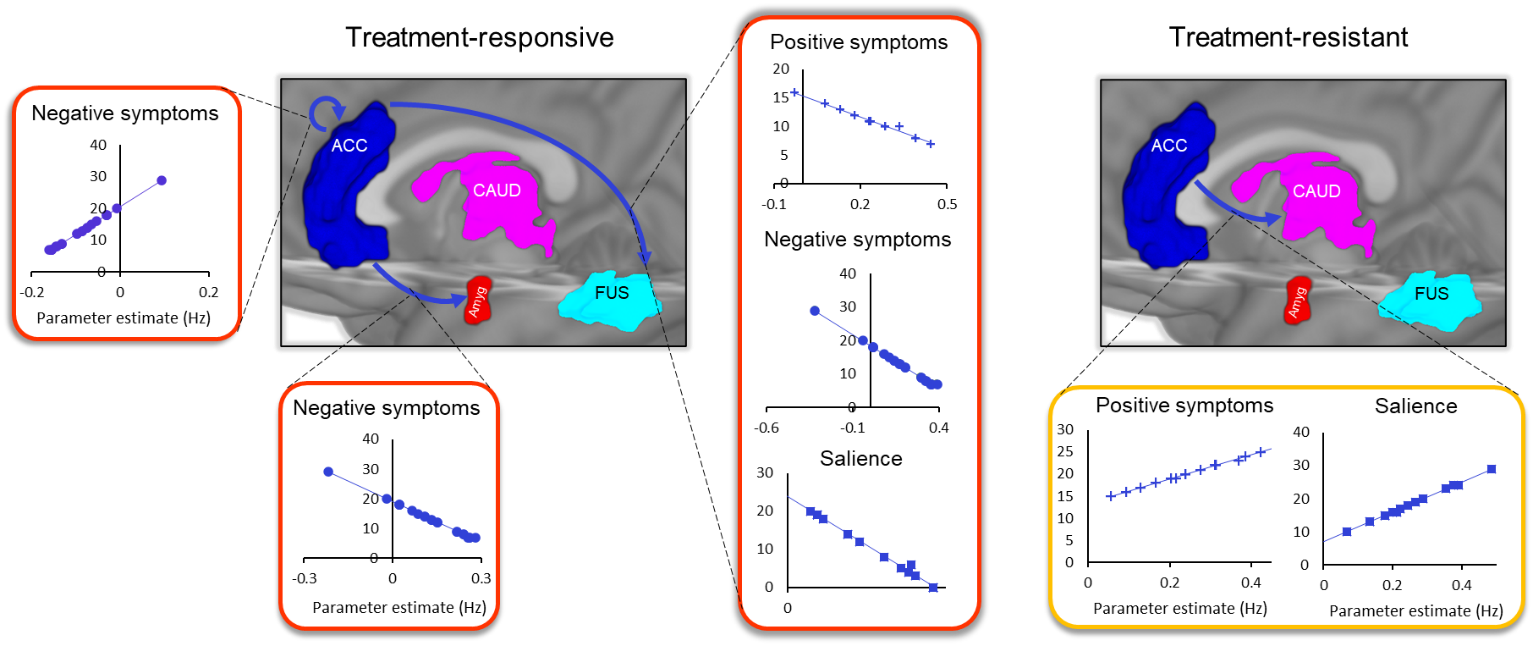
**

**Figure S4. Top-down connectivity related to symptoms and salience.**

Complementary figure to Figure 3 in the main text, again showing the top-down connections (from ACC) that show significant relationships with positive symptoms, negative symptoms and salience scores using Parametric Empirical Bayes (PEB) for (A) responsive and (B) resistant patients (Pp > 0.95). In this figure, the parameter estimates (based on a prior covariate) are correlated back against symptom scores (and are not Pearson correlation plots).

**
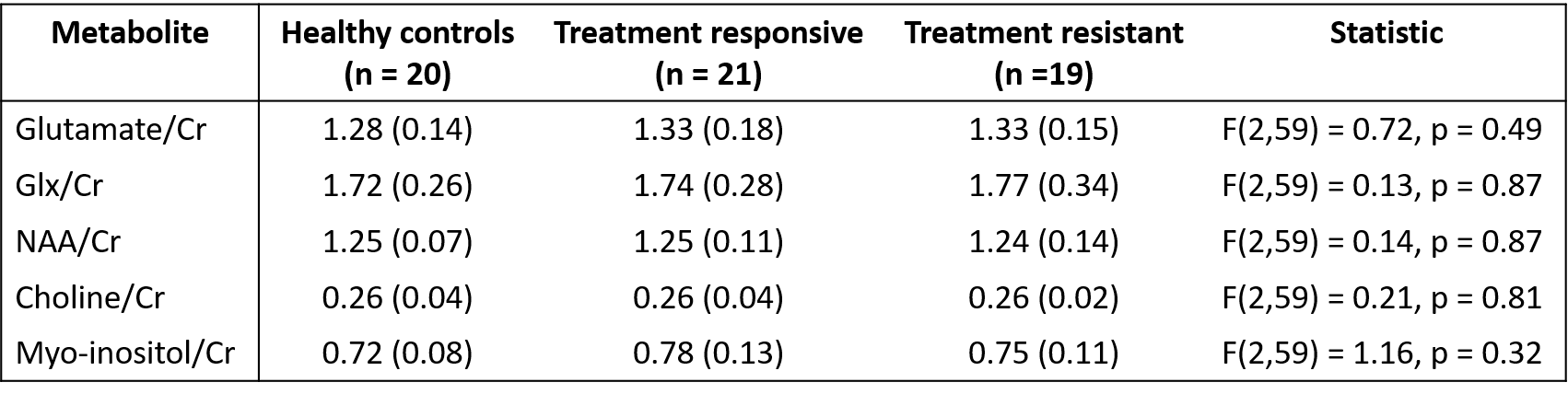
Table S3. Anterior Cingulate Cortex metabolite level ratios as measured using 1H-MRS.**

*Note:* Values are mean (SD) and statistics from One-way ANOVA. Cr = Creatine; Glx = Glutamate plus Glutamine; NAA = N-acetylaspartate.

**Table S4. PEB results for group x glutamate interaction.**

| Fixed connections | Responsive > HC | | Resistant > HC | | Resistant > Responsive | |
| --- | --- | --- | --- | --- | --- | --- |
|  | Effect size (Hz) | Pp | Effect size (Hz) | Pp | Effect size (Hz) | Pp |
| ACC 🡪 Fus | 0.01 | 0.55 | **-0.049** | **1** | **-0.062** | **1** |
| ACC 🡪 Caud | **-0.057** | **1** | 0.026 | 0.90 | **0.082** | **1** |
| ACC 🡪 Amyg | 6.35E-06 | 0 | -0.031 | 0.947 | **-0.032** | **0.95** |
| ACC 🡪 ACC | 0.023 | 0.83 | 0.031 | 0.94 | -2.02E-06 | 0 |

*Note:* Three PEB models showing the parameter estimates and associated Posterior probabilities (Pp) for the group interaction on the parametric effect of glutamate. Only the top-down connections were examined, and significant connections are shown in bold (Pp > 0.95).

**Further references:**

1. J. D. Power, K. A. Barnes, A. Z. Snyder, B. L. Schlaggar, S. E. Petersen, Spurious but systematic correlations in functional connectivity MRI networks arise from subject motion. *NeuroImage* **59**, 2142-2154 (2012).

2. L. D. Vanes, E. Mouchlianitis, T. Collier, B. B. Averbeck, S. S. Shergill, Differential neural reward mechanisms in treatment-responsive and treatment-resistant schizophrenia. *Psychol Med* **48**, 2418-2427 (2018).

3. K. J. Friston, L. Harrison, W. Penny, Dynamic causal modelling. *Neuroimage* **19**, 1273-1302 (2003).

4. W. D. Penny, K. E. Stephan, A. Mechelli, K. J. Friston, Comparing dynamic causal models. *Neuroimage* **22**, 1157-1172 (2004).

5. P. Zeidman *et al.*, A guide to group effective connectivity analysis, part 2: Second level analysis with PEB. *Neuroimage* **200**, 12-25 (2019).
